# Basal-bolus insulin therapy in postoperative inpatients with diabetes mellitus: directions for future quality-improvement initiatives

**DOI:** 10.4155/fsoa-2017-0099

**Published:** 2017-10-25

**Authors:** Curtiss B Cook, Heidi A Apsey, Amy E Glasgow, Janna C Castro, Elizabeth B Habermann, Richard T Schlinkert

**Affiliations:** 1 Division of Endocrinology, Mayo Clinic, Scottsdale, AZ, USA; 2 Division of General Surgery, Mayo Clinic Hospital, Phoenix, AZ, USA; 3 Department of Information Technology, Mayo Clinic Hospital, Phoenix, AZ, USA; 4 Division of Health Care Policy & Research & Robert D & Patricia E Kern Center for Science of Health Care Delivery, Mayo Clinic, Rochester, MN, USA

**Keywords:** endocrinology, metabolism, surgery

## Abstract

**Aim:**

To determine variables associated with hyperglycemia and insulin therapy in postoperative inpatients with diabetes mellitus following a quality-improvement initiative.

**Materials & methods:**

Patients with diabetes mellitus following an elective surgical procedure (n = 782; 877 surgical procedures) were selected.

**Results:**

Age, hemoglobin A_1c_ corticosteroids, insulin therapy and year of surgery were associated (p < 0.01) with hyperglycemia. Hemoglobin A_1c_, hyperglycemia, case mix index and corticosteroids were associated (p ≤ 0.03) with insulin therapy. Hyperglycemia and use of insulin varied by surgical specialty.

**Conclusion:**

Data could be used to modify current treatment algorithms. Variations in hyperglycemia and insulin use by surgical specialty require further investigation.

## Lay abstract

It is important to control blood sugar levels in the hospital in patients who have had surgery. We found that certain characteristics such as hemoglobin A_1c_, disease complexity, frequency of high sugar measurements and use of steroids predicted the need for insulin. We also found that insulin was provided inconsistently across surgical specialties. These findings from this analysis could be used to develop uniform therapy to improve outcomes for surgical patients with diabetes mellitus.

The relationship between hyperglycemia and adverse outcomes in postoperative patients in the hospital is well documented. Adverse outcomes include increased incidence of surgical-site wound infections, longer length of stay, higher mortality rate and greater frequency of reoperative interventions [1–7]. Available data indicate that treating hyperglycemia can lower the risk of complications related to poor inpatient glucose control [8–12]. Current guidelines promote a target glucose range of 140–180 mg/dl for critically and noncritically ill inpatients [13–15].

The most effective approach for managing hyperglycemia in hospitalized noncritically ill patients, including postoperative patients, is basal-bolus insulin therapy. If the patient is eating, this treatment consists of a combination of long- or intermediate-acting insulin with short- or rapid-acting insulin given with meals, supplemented with correction doses [12–18]. In patients who are not eating, basal insulin combined with a correction dose is also effective [19]. Correction insulin alone – the so-called sliding scale – does not provide sufficient control of hyperglycemia [20,21]. Because hypoglycemia can be associated with greater inpatient mortality, the basal-bolus insulin treatment approach can provide the best balance between controlling hyperglycemia while decreasing the risk of hypoglycemia [22].

We previously identified basal-bolus insulin therapy as underutilized in postoperative patients with diabetes mellitus (DM), despite evidence of ongoing hyperglycemia [23]. To overcome this clinical inertia in basal-bolus insulin use, we developed a quality-improvement initiative for our hospital and implemented it in 2012. This initiative was designed to increase the postoperative administration of basal-bolus insulin therapy to noncritically ill patients. A preliminary analysis showed marked improvement in the administration of basal-bolus insulin therapy [12]. Specifically, the administration of basal-bolus insulin therapy increased from 9% of cases prior to intervention to 32% after the performance improvement project was initiated [12]. A follow-up analysis indicated that this increase in use, while sustained over a 4-year period, plateaued and did not improve beyond what was seen immediately after the intervention was implemented [24]. To identify specific factors that might guide future quality-improvement initiatives (i.e., further improve basal-bolus insulin use and decrease the incidence of hyperglycemia), we conducted an analysis of the available data to determine factors correlated with glucose control and basal-bolus insulin therapy.

## Methods

### Description of intervention

The approach used to increase the use of basal-bolus insulin therapy has been detailed in prior publications. A quality-improvement initiative was developed from 1 April 2012, to 31 May 2012, in response to data indicating the suboptimal use of basal-bolus insulin therapy in postoperative patients [12,23]. The initiative, designed to increase the use of basal-bolus insulin therapy in noncritically ill inpatients, targeted residents, faculty members and advanced level practitioners in nine surgical services (colorectal, general, gynecologic, neurologic, orthopedic, otolaryngologic, plastic urologic, and vascular surgery). A care process model was developed and was provided during educational sessions [12,25]. The model provided guidance on when to use basal-bolus insulin therapy. For instance, if the patient was receiving insulin as an outpatient, continued postoperative insulin administration was recommended, and basal-bolus insulin therapy was encouraged for insulin-naive patients if two glucose measurements >180 mg/dl were recorded within 24 h after surgery [12,25]. A nurse practitioner monitored cases and provided advice to support the surgical services. The project was approved by the Institutional Review Board.

### Case selection & data extraction

The methods for case selection and data extraction were previously published [12]. Briefly, patients selected from the electronic health records had a known diagnosis of DM, had undergone an elective surgical procedure under general anesthesia from 1 June 2012, through 9 February 2015 and were hospitalized postoperatively. Demographic variables, outpatient DM therapy, hemoglobin A_1c_ (HbA_1c_), type of surgical service and data on point-of-care blood glucose (POC-BG) levels and insulin therapy were obtained [12]. The case mix index was used to adjust for differences in disease complexity based on costs. The case mix index uses the weights for the Medicare Severity–Diagnosis Related Group assigned by the Centers for Medicare and Medicaid Services [26]. Surgical services were categorized as general, orthopedic, urologic, neurosurgical, otolaryngologic or other (e.g., services with a low frequency of DM patients such as gynecologic and plastic surgery were combined). Cardiothoracic patients were not included because a separate protocol for tight glycemic control is used for these patients [12].

### Assessment of insulin therapy

Only insulin administered to the patient was included in the analysis. Insulin treatment regimens were categorized as previously defined [12,23–24]. Long- and intermediate-acting insulin therapy was classified as ‘basal’, and rapid- and short-acting insulin was classified as ‘short-acting’ if it was provided as a prandial dose, correction dose or both. Patterns of insulin therapy were stratified as ‘none’, ‘basal only’, ‘short-acting only’, or ‘basal plus short-acting’. Any use of premixed insulin was classified as basal plus short-acting, if its use was identified. As in previously published studies, the analyses were restricted to patients with a length of stay of 3 days or longer [12,23–24]. This allowed time for changes in insulin therapy to occur.

### Statistical analysis

Regression models were constructed to examine the variables associated with the incidence rates for hyperglycemia (defined as >180 mg/dl glucose) and hypoglycemia (<70 mg/dl glucose) and for the use of basal-bolus insulin therapy. Outpatient DM therapy did not contribute to the models and hence was not included. Generalized estimating equation (GEE) models were used to adjust for differences and account for repeated measures (i.e., more than one hospitalization per patient). Poisson regression was used to model the percentage of POC-BG measurements per patient that showed hyperglycemia or hypoglycemia. The per-patient frequency of hyperglycemia was calculated by dividing the number of measurements >180 mg/dl by the total number of measurements taken. Similarly, the number of values <70 mg/dl was divided by the total number of POC-BG measurements to calculate the per-patient frequency of hypoglycemia. The dependent variable in each model was the percentage of measurements, and the offset variable was the denominator of each measure. The distributions of the measurements were checked to verify the Poisson distributions. The data analysis was performed using SAS 9.4 (SAS Institute, Inc. Cary, NC, USA). Patients with missing HbA_1c_ data (n = 77) were excluded from the final analysis.

## Results

### Patient characteristics

The final dataset consisted of 782 patients who underwent 877 surgical procedures (128 performed in 2012; 320 in 2013; 388 in 2014; 41 in 2015) from 1 June 2012, through 9 February 2015; 695 patients underwent one procedure, 79 underwent two and 8 underwent three. The mean (SD) age was 66 [11] years, most patients were men (56%), and most patients were white (89%; Table 1). Their preoperative metabolic control, as determined by their HbA_1c_, was good. Most patients were managed with oral agents as outpatients. The majority of  procedures were classified as general, orthopedic or urologic. Less than half the patients received glucocorticoids while hospitalized. The mean (SD) prevalence of hyperglycemia (as measured by POC-BG tests) was 18% (21%) of total measurements, but hypoglycemia was rare (1.1% [4.2%]; Table 1).

**Table 1.** **Patient characteristics (n = 877 procedures in 782 patients)^†^.**

**Characteristic**	**Number**	**Value^‡^**
Age, years	877	66 (11)

**Sex**		

– Men	491	56

**Race/ethnicity**		

– White	778	89

HbA_1c_, %	877	6.7 (1.0)

**Outpatient insulin therapy**		

– Diet	114	13

– Insulin only	148	17

– Insulin and oral agents	202	23

– Oral agents only	407	46

– Other	6	1

Case mix index	877	2.42 (1.19)

**Surgical service**		

– General surgery	317	36

– Orthopedics	290	33

– Urology	131	15

– Otolaryngology	52	6

– Other	46	5

– Neurosurgery	41	5

Received corticosteroids	313	36

**Glucose status, % of blood tests**		

– Hyperglycemia (>180 mg/dl)		18 (21)

– Hypoglycemia (<70 mg/dl)		1.1 (4.2)

^†^In total, 782 patients with diabetes mellitus underwent 877 surgical procedures.

^‡^Values are presented as the mean (SD) or percentage.

HbA_1c_: Hemoglobin A_1c_.

### Variables associated with hyperglycemia & hypoglycemia

Using GEE methods, a linear regression model was constructed to examine the variables associated with hyperglycemia (Table 2). After adjusting for other variables, age, HbA_1c_, type of surgical service, use of glucocorticoids, use of basal-bolus insulin therapy and year of surgery were significantly associated with hyperglycemia frequency. Every 5-year increase in age was associated with a 3% (95% CI: 1.02–1.04) increase in the hyperglycemia frequency, and every 1% increase in HbA_1c_ was correlated with an 18% (95% CI: 1.15–1.21) increase in hyperglycemia frequency. Neurosurgical patients had a 52% (95% CI: 1.34–1.71) higher hyperglycemia frequency compared with general surgery patients. However, orthopedic patients and patients treated by other surgical services had 8% and 23% lower frequencies, respectively, compared with general surgery, and the use of glucocorticoids led to an 8% (95% CI: 1.08–1.12) increase in glucose measurements >180 mg/dl. Basal-bolus insulin therapy was associated with a 49% (95% CI: 1.44–1.59) increase in hyperglycemia frequency. Compared with patients treated in 2012, those treated in 2014 were characterized by a lower frequency of hyperglycemia, but patients treated in 2015 were associated with a 35% higher rate of hyperglycemia.

**Table 2.** **Linear regression analysis showing variables associated with the frequency of postoperative inpatient hyperglycemia^†^.**

**Factor**	**β**	**Lower 95% CL**	**Upper 95% CL**	**p-value**
Age, years^‡^	1.03	1.02	1.04	<0.001

Female sex	0.99	0.96	1.02	0.47

**Race/ethnicity**				

– White	1.00	0.95	1.04	0.91

HbA_1c_ %	1.18	1.15	1.21	<0.001

Case mix index	0.99	0.97	1.01	0.32

**Surgical service**^§^				

– Neurosurgery	1.52	1.34	1.71	<0.001

– Orthopedics	0.92	0.86	0.99	0.02

– Other	0.77	0.68	0.87	<0.01

– Otolaryngology	0.97	0.88	1.06	0.51

– Urology	1.06	0.98	1.15	0.16

Received corticosteroids	1.08	1.04	1.12	<0.001

Basal-bolus insulin^¶^	1.49	1.44	1.59	<0.001

**Year of surgery**^#^				

– 2013	0.99	0.90	1.10	0.90

– 2014	0.87	0.79	0.96	0.004

– 2015	1.35	1.17	1.56	<0.01

^†^In total, 782 patients with diabetes mellitus underwent 877 surgical procedures.

^‡^Per 5-year increase.

^§^Compared with general surgery.

^¶^Compared with no basal-bolus insulin.

^#^Compared with 2012.

CL: Confidence limit; HbA_1c_: Hemoglobin A_1c_.

Given the low frequency of hyperglycemia, the risk of hypoglycemia was assessed using GEE logistic regression analysis with any instance of hypoglycemia used as the outcome (Table 3). For every 5-year increase in age, the chance of a hypoglycemic episode increased by 13% (95% CI: 1.03–1.25). The use of basal-bolus insulin therapy increased the odds over fivefold (95% CI: 3.25–8.92). No other variable, including year of procedure, contributed to hypoglycemia risk.

**Table 3.** **Logistic regression showing the variables associated with risk of postoperative hypoglycemia^†^.**

**Factor**	**Odds ratio**	**Lower 95% CL**	**Upper 95% CL**	**p-value**
Age, years^‡^	1.13	1.03	1.25	0.01

Female sex	0.81	0.51	1.29	0.38

White	0.72	0.38	1.37	0.31

HbA_1c_ %	1.01	0.81	1.26	0.92

Case mix index	1.09	0.93	1.28	0.29

**Surgical service**^§^				

– Neurosurgery	0.30	0.07	1.38	0.12

– Orthopedics	0.57	0.33	0.99	0.05

– Other	1.18	0.44	3.17	0.74

– Otolaryngology	1.08	0.46	2.52	0.87

– Urology	0.83	0.43	1.59	0.57

Received corticosteroids	1.07	0.66	1.73	0.79

Basal-bolus insulin	5.38	3.25	8.92	<0.001

**Year of surgery**^¶^				

– 2013	0.94	0.48	1.83	0.86

– 2014	1.06	0.56	2.01	0.86

– 2015	0.72	0.21	2.45	0.60

^†^In total, 782 patients with diabetes mellitus underwent 877 surgical procedures.

^‡^Per 5-year increase.

^§^Compared with general surgery.

^¶^Compared with 2012.

CL: Confidence limit; HbA_1c_: Hemoglobin A_1c_.

### Variables associated with basal-bolus insulin therapy

Next, the characteristics associated with basal-bolus insulin therapy were evaluated using GEE logistic regression analysis (Table 4). After adjusting for the other variables in Table 3, HbA_1c_ (p < 0.01), hyperglycemia frequency (p < 0.01), case mix index (p < 0.01), type of surgical service and use of glucocorticoids (p < 0.01) were significantly associated with basal-bolus insulin therapy. Every 1% increase in HbA_1c_ was associated with a 300% (95% CI: 2.35–3.87) increase in the odds of using basal-bolus insulin therapy, and every 10% increase in the incidence of hyperglycemia increased the chance of administering basal-bolus insulin therapy by 50% (95% CI: 1.35–1.66). Every unit increase in the case mix index also increased the odds of administering basal-bolus therapy by 18% (95% CI: 1.10–1.36).

**Table 4.** **Logistic regression showing the variables associated with the use of inpatient basal-bolus insulin therapy^†^.**

**Factor**	**Odds ratio**	**Lower 95% CL**	**Upper 95% CL**	**p-value**
Age, years^‡^	0.95	0.87	1.03	0.22

Female sex	0.84	0.57	1.25	0.39

White	0.78	0.43	1.38	0.39

HbA_1c_ %	3.01	2.35	3.87	<0.001

Blood glucose >180 mg/dl, %^§^	1.50	1.35	1.66	<0.001

Case mix index	1.18	1.01	1.36	0.03

**Surgical service**^¶^				

– Neurosurgery	0.13	0.04	0.40	<0.001

– Orthopedics	0.50	0.31	0.80	0.004

– Other	0.51	0.19	1.33	0.17

– Otolaryngology	1.08	0.49	2.40	0.85

– Urology	0.48	0.27	0.87	0.02

Received corticosteroids	1.90	1.25	2.87	0.003

**Year of surgery**^#^				

– 2013	0.79	0.45	1.39	0.41

– 2014	0.81	0.47	1.39	0.43

– 2015	0.44	0.16	1.21	0.11

^†^In total, 782 patients with diabetes mellitus underwent 877 surgical procedures.

^‡^Per 5-year increase.

^§^Per 10% increase.

^¶^Compared with general surgery.

^#^Compared with 2012.

CL: Confidence limit; HbA_1c_: Hemoglobin A_1c_.

The surgical services did not consistently administer basal-bolus insulin therapy. The odds of basal-bolus insulin therapy use by the neurosurgery service was 87% less than general surgery, and in orthopedics and urology, the chances were 50 and 52% lower, respectively. Administering glucocorticoid therapy increased the odds of administering basal-bolus insulin therapy by 90% (95% CI: 1.25–2.87; Table 3). Finally, the chances of administering basal-bolus insulin therapy decreased with time, such that its administration was 56% lower by 2015 compared with 2012.

### Assessment of insulin therapy progression

**Figure 1.**
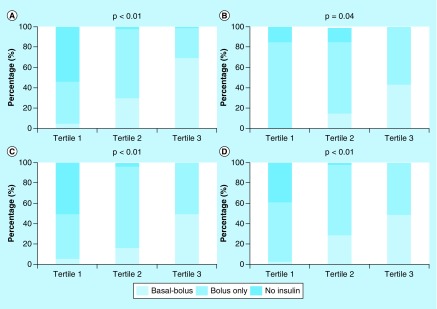
**Changes in insulin regimen by the tertiles of percentage of point-of-care blood glucose measurements >180 mg/dl.** **(A)** General surgery (referent). **(B)** Neurosurgery. **(C)** Orthopedics. **(D)** Urology.

We further analyzed how therapy was administered with respect to hyperglycemia after observing that less basal-bolus insulin therapy was administered in the neurosurgical, orthopedic and urologic services compared with general surgery. The proportion of patients who received basal-bolus insulin treatment was plotted against the frequency (by tertile) of POC-BG measurements >180 mg/dl (Figure 1) [12,23–24]. The referent surgical service (general surgery) showed a significant progression in the use of basal-bolus insulin therapy with the increasing frequency of hyperglycemic measurements: 69% of patients in the highest tertile for hyperglycemic episodes received basal-bolus insulin therapy (Figure 1A). A significant intensification of therapy, resulting in the greater use of basal-bolus insulin, also took place in patients treated in the neurosurgical (Figure 1B), orthopedic (Figure 1C) and urologic specialties (Figure 1D). However, unlike the patients on general surgery, only 43% of neurosurgery patients, 49% of orthopedics patients and 48% of urology patients whose hyperglycemia frequencies were in the highest tertile received basal-bolus insulin therapy.

## Discussion

Optimizing glycemic control in postoperative inpatients can reduce associated complications [8–11]. Current standards for treating noncritically ill inpatients encourage implementing basal-bolus insulin therapy for hyperglycemia to achieve the recommended glucose targets [13–15]. Our previous quality-improvement initiative showed an increase in the use of basal-bolus therapy in the months after implementation, although therapy plateaued and did not further improve with time [12,24]. To provide some guidance about how future quality improvements could be directed, this analysis was performed to more specifically identify variables that might be associated with glucose control and basal-bolus insulin therapy. Additionally, this analysis provides specific data on individual surgical services.

In this aggregate analysis of data collected from 1 June 2012, through 9 February 2015, older age, HbA_1c_ and the use of glucocorticoids were all associated with increased hyperglycemia frequency. This information could be used by surgical teams to recognize the higher probability of hyperglycemia in patients with these characteristics. Current institutional treatment algorithms could be modified to account for these variables and facilitate the earlier administration of basal-bolus insulin therapy. The finding that the hyperglycemia frequency was higher in 2015 compared with 2012 could be concerning and justifies the need for continued data collection, analysis and staff education about postoperative DM care. The results help identify which surgical teams could receive more intensive effort and education regarding the management of inpatient hyperglycemia. For instance, a higher hyperglycemia frequency was detected in patients treated by neurosurgery compared with general surgery, but a lower hyperglycemia frequency was detected in patients treated by orthopedics and other surgical services. Neurosurgical practitioners may need more intensive education and training regarding the techniques that improve glucose control.

A prior study reported that hypoglycemic events did not change in the analysis period after implementing our quality-improvement initiative [24]. In the current analysis, the risk of hypoglycemia increased with age and the use of basal-bolus insulin therapy. Basal-bolus insulin therapy is a more intensive treatment program that can be associated with greater hypoglycemia risk. Hospitals need to have a policy and procedure in place for recognizing and treating hypoglycemia, particularly if there are plans to institute more intensive insulin therapy.

After adjusting for other variables, increased HbA_1c_ and hyperglycemic measurements and corticosteroid use were associated with a greater chance of basal-bolus insulin therapy being administered. Basal-bolus insulin therapy was also correlated with a greater risk of hyperglycemia.  However, when considering these results of Tables 2 and 4, these findings indicate that clinicians were responding to higher glucose levels with greater use of insulin. A higher case mix index also increased the odds of administering basal-bolus insulin. Because the case mix index is a measure of patient complexity, this finding does suggest that sicker patients will likely need basal-bolus insulin therapy to manage their glucose levels. As with predictors of hyperglycemia, these data can be used to refine the treatment algorithms used to assist with decision making in situations when basal-bolus insulin therapy would be appropriate.

All surgical services received the same guidelines and ancillary support from the nurse practitioner during the quality-improvement initiative, so the administration of basal-bolus insulin therapy should have been uniform across the specialties. However, as with variations in hyperglycemia frequency, differences were detected in the use of basal-bolus insulin therapy according to surgical service. The odds of receiving basal-bolus insulin therapy were lowest among neurosurgical, orthopedic and urologic patients compared with general surgery. Differences in hyperglycemia between surgical services could be explained by not only whether basal-bolus insulin therapy was used but also by differences in the amplitudes and frequencies of the changes in insulin doses. For instance, orthopedic patients had less hyperglycemia than general surgery patients, but they also had lower odds of receiving basal-bolus insulin therapy. These incongruent findings could be explained if, compared with other practices, the orthopedic teams increased the insulin doses more aggressively or more often in patients receiving basal-bolus insulin therapy. More details about the insulin-ordering practices of the individual surgical services are needed. Although not statistically significant, the odds of receiving basal-bolus insulin therapy decreased by 2015, which may explain why the frequency of hyperglycemia was higher that year compared to 2012 as detected in the regression analysis. The lower use of basal-bolus insulin in 2015, and the greater frequency of hyperglycemia could be evidence of the need to go revisit how inpatient hyperglycemia treatment strategies are being taught.

This analysis has some limitations. First, the conclusions are based on an analysis of retrospective data. Second, the data do not allow assessment of clinician-specific decision making. Direct observation of, and immediate feedback to, clinicians regarding the decision to administer (or not administer) insulin therapy would be helpful. Third, the data are derived from a single institution and cannot be generalized to other facilities. Finally, there is selection bias regarding metabolic control. The patients included in this analysis had good glycemic control, with an average HbA_1c_ of 6.7%. Another aspect of the quality-improvement initiative was that  patients with >8% HbA_1c_ would have elective surgery postponed until glycemic control improved, thus skewing the analysis to cases with better preoperative glucose control. Including patients with poorer metabolic control could alter the results of the analysis.

## Conclusion

Nonetheless, the data provide insights on the care and management of inpatient hyperglycemia in patients with DM after implementing a quality-improvement initiative for surgical practices. Like outpatient DM therapy, inpatient DM treatment will need to be individualized. The variables identified here that were associated with hyperglycemia or predictive of the use of basal-bolus insulin therapy could be used to construct guidelines on modifying treatment algorithms for surgical practitioners. For instance, an equation based on logistic models could be used to estimate the need to start basal-bolus insulin therapy. The basis for the variations in hyperglycemia and insulin use according to the type of surgical service requires further investigation to ensure that a unified treatment strategy is applied to all surgical patients.

## Future perspective

Despite the importance of basal-bolus insulin therapy for hospitalized patients with DM, very little is actually known about what factors drive therapy. Our analysis provides some insights into some of the variables that predict treatment, but additional studies would shed light on this topic. For instance, it would be interesting to determine if the same variables identified here that predict the use of basal-bolus insulin therapy are present in other institutions. The factors found here (e.g., higher HbA1c percent, more frequent hyperglycemia, illness complexity, corticosteroid therapy) could be common drivers for treatment elsewhere. There are very little data on what influences practitioners’ decisions to intensify insulin therapy in the hospital. A real-time survey (i.e., while patients are hospitalized) could be conducted to ask clinicians why they did (or did not) change treatment when faced with evidence of persistent hyperglycemia.

Additionally, there is a paucity of information about other aspects of inpatient insulin therapy such as the amplitude and frequency of dosing changes, and these parameters impact the efficacy of controlling glucose levels. Data on insulin–glucose, dose–response relationships in dynamic hospital settings are lacking. Finally, better guidelines are needed on selecting the best candidates for basal-bolus insulin therapy. For instance, current guidelines do not distinguish between patients who have only intermittent or mild hyperglycemia and patients who have higher hyperglycemia values or a greater frequency of hyperglycemic events [14,15].

**Summary points**

- An analysis was conducted to determine factors correlated with glucose control and basal-bolus insulin therapy in postoperative inpatients with diabetes mellitus.
- Data on 877 surgical procedures were obtained from the electronic health records, and regression analyses were performed using generalized estimating equations to evaluate the variables associated with hyperglycemic frequency and basal-bolus insulin therapy.
- Age, hemoglobin A_1c_, use of corticosteroids, use of basal-bolus insulin therapy and year of surgery were associated (all p < 0.01) with hyperglycemia frequency in the adjusted analysis.
- Hemoglobin A_1c_, hyperglycemia frequency, case mix index and the use of corticosteroids were associated (all p ≤ 0.03) with basal-bolus insulin therapy.
- Hyperglycemia frequency and the use of basal-bolus insulin therapy varied by surgical specialty.
- Information from this analysis can be used to modify current treatment decision algorithms.
- Variations in hyperglycemia frequency and insulin use according to surgical specialty require further investigation.
